# Canadian Dairy Network for Antimicrobial Stewardship and Resistance (CaDNetASR): An On-Farm Surveillance System

**DOI:** 10.3389/fvets.2021.799622

**Published:** 2022-01-12

**Authors:** Mariana Fonseca, Luke C. Heider, David Léger, J. Trenton Mcclure, Daniella Rizzo, Simon Dufour, David F. Kelton, David Renaud, Herman W. Barkema, Javier Sanchez

**Affiliations:** ^1^Health Management Department, University of Prince Edward Island, Charlottetown, PE, Canada; ^2^Public Health Agency of Canada, Center for Foodborne, Environmental and Zoonotic Infectious Diseases, Guelph, ON, Canada; ^3^Department of Pathology and Microbiology, Faculty of Veterinary Medicine, Université de Montréal, Saint-Hyacinthe, QC, Canada; ^4^Department of Population Medicine, Ontario Veterinary College, University of Guelph, Guelph, ON, Canada; ^5^Department of Production Animal Health, University of Calgary, Calgary, AB, Canada

**Keywords:** dairy cattle, antimicrobial use, antimicrobial resistance, surveillance, Canada

## Abstract

Canada has implemented on-farm antimicrobial resistance (AMR) surveillance systems for food-producing animals under the Canadian Integrated Program for Antimicrobial Resistance (CIPARS); however, dairy cattle have not been included in that program yet. The objective of this manuscript was to describe the development and implementation of the Canadian Dairy Network for Antimicrobial Stewardship and Resistance (CaDNetASR). An Expert Panel (EP) of researchers was created to lead the development of the dairy surveillance system. The EP initiated a draft document outlining the essential elements of the surveillance framework. This document was then circulated to a Steering Committee (SC), which provided recommendations used by the EP to finalize the framework. CaDNetASR has the following components: (1) a herd-level antimicrobial use quantification system; (2) annually administered risk factor questionnaires; and (3) methods for herd-level detection of AMR in three sentinel enteric pathogens (generic *Escherichia coli, Campylobacter* spp., and *Salmonella* spp.) recovered from pooled fecal samples collected from calves, heifers, cows, and the manure pit. A total of 144 dairy farms were recruited in five Canadian provinces (British-Columbia, Alberta, Ontario, Québec, and Nova-Scotia), with the help of local herd veterinarians and regional field workers, and in September 2019, the surveillance system was launched. 97.1 and 94.4% of samples were positive for *E. coli*, 63.8, and 49.1% of samples were positive for *Campylobacter* spp., and 5.0 and 7.7% of samples were positive for *Salmonella* spp., in 2019 and 2020, respectively. *E. coli* was equally distributed among all sample types. However, it was more likely that *Campylobacter* spp. were recovered from heifer and cow samples. On the other hand, it was more common to isolate *Salmonella* spp. from the manure pit compared to samples from calves, heifers, or cows. CaDNetASR will continue sampling until 2022 after which time this system will be integrated into CIPARS. CaDNetASR will provide online access to farmers and veterinarians interested in visualizing benchmarking metrics regarding AMU practices and their relationship to AMR and animal health in dairy herds. This will provide an opportunity to enhance antimicrobial stewardship practices on dairy farms in Canada.

## Introduction

Antimicrobial resistance (AMR) is a natural phenomenon that occurs when bacteria evolve and no longer respond to antimicrobial drugs that previously were efficacious. Major economic losses and animal health and welfare problems have been described as the consequences of AMR (1, 2). Many AMR commensal and pathogenic bacteria have been described in food animals. For instance, a study conducted in North California demonstrated that all *Salmonella* Newport isolates recovered from dairy cattle fecal samples (symptomatic and asymptomatic animals) were multidrug-resistant (3). Infections caused by *Salmonella* Newport can cause economic losses due to treatment failure and increase mortality rates in animals (4). Many bacterial organisms, including *Salmonella* Newport can be shared between human and animal populations. In humans, AMR can make treatment of bacterial infections more challenging, increase treatment costs, allow for increased disease spread, and increase the risk of mortality in people (5). It is estimated that 700,000 deaths worldwide are caused annually by antimicrobial resistant bacteria and, by 2050, this figure may increase to 10 million (6). For these reasons, AMR is considered one of the major challenges to public health (7).

To address the global problem of AMR, many countries have developed and implemented AMR surveillance systems for humans and animals. A surveillance system can be defined as “a system based on continuous information recording, making it possible to monitor the health status of a given population and the risk factors to which it is exposed, to detect pathological processes as they appear and study their development in time and space, and then to take appropriate measures to control them” (8). The main objectives of an on-farm AMR surveillance system are: (1) to determine the current prevalence of AMR (2) to describe AMR trends; (3) to detect the emergence of new types of resistance; and (4) to track a particular type of resistance (9).

In addition, this surveillance system should be able to provide estimates of the types and amount of antimicrobials used on farms. Evidence (6) suggests associations between using certain antimicrobials in animals with resistance in clinical bacterial isolates from humans (10). Similar to the situation in humans, there is also a strong association between antimicrobial use (AMU) and AMR in the livestock sector (11–14). In the dairy sector, the route of administration and the antimicrobial active ingredient seem to play an important role in the development of antimicrobial resistance. A study conducted in Canada demonstrated that the use of systemic antimicrobials was associated with resistance in non-aureus staphylococci isolated from milk, while intramammary treatments were not (15). However, a study conducted in Ohio found that the use of cephalosporin based dry cow therapy was associated with recovering a greater number of fecal coliform bacteria with reduced susceptibility to cephalothin and streptomycin in dairy cows (16).

Recognizing the interrelationship between AMU/AMR in humans and animals and the need for the standardization of methods between countries (e.g., AMU metrics, target pathogens, etc.), in 2018, the Food and Agriculture Organization of the United Nations (FAO), the World Organization for Animal Health (OIE), and the World Health Organization (WHO) formed a tripartite alliance (FAO-OIE-WHO) focusing on a “One Health” approach to AMR (17). The “One Health” approach includes surveillance of important AMR organisms and AMU in humans, animals, and the environment.

In support of this One Health approach to AMR, many countries developed surveillance systems to monitor AMU and AMR in food animal agriculture (15). Many of these surveillance systems report the proportion of antimicrobial resistant isolates of *Salmonella* spp., *Campylobacter* spp., and *Escherichia coli* (8), as these pathogens can be transmitted zoonotically through the food chain to humans.

Denmark and the Netherlands have comprehensive AMU surveillance systems (DANMAP and Nethmap-MARAN, respectively) (18). In Canada, the Canadian Integrated Program for Antimicrobial Resistance Surveillance (CIPARS) was developed in 2002 to collect and analyze AMU/AMR data, and report trends in AMU and AMR from human, retail food, and food-producing animals (19). In 2006, CIPARS implemented an on-farm component in grower-finisher pigs; then, in 2013, in broiler chicken and turkey (20), and in 2019, a surveillance system for feedlot cattle was started (21). These national surveillance systems collect AMU data at the farm level to facilitate AMU benchmarking for farms and for developing interventions toward antimicrobial stewardship (AMS).

Reducing AMU in humans and animals is crucial to diminish the burden of AMR and prolong antimicrobial efficacy (22). In Canada, initiatives led by the Canadian Veterinary Medical Association (CVMA) and the Public Health Agency of Canada (PHAC) have created guidelines to improve AMS. The CVMA defines AMS as “multifaceted and dynamic approaches required to sustain clinical efficacy of antimicrobials.” In 2017, the PHAC released the document “*Tackling Antimicrobial Resistance and Antimicrobial Use: A Pan-Canadian Framework for Action.”* The framework's goal was to strengthen the ability to fight AMR in a coordinated, multisectoral and effective manner (23). AMS was one of the components promoted to achieve the goal. However, despite these initiatives, there are still challenges because the coordination of AMS leadership is sparse and inconsistent across the country (23).

In the dairy sector, some factors, such as dairy consumer perception, government requirements, and animal and human health are the main reasons for continuing to work on AMS programs (24). Recognizing the knowledge gap on AMR and AMU in the dairy sector in Canada, the Canadian Dairy Network for Antimicrobial Stewardship and Resistance (CaDNetASR) was developed to help determine and improve AMU stewardship on Canadian dairy farms. This surveillance system will estimate AMU, determine how and why antimicrobials are used on dairy farms, and determine AMR patterns and trends in the Canadian dairy sector. This manuscript aims to describe the development and implementation of a national on-farm surveillance system (CaDNetASR), for an ongoing AMU and AMR data collection on Canadian dairy farms, toward improved AMS in this production sector.

## CaDNetASR Surveillance Framework Development and Implementation

Research personnel from five veterinary colleges in Canada (University of Prince Edward Island, University of Guelph, University of Saskatchewan, University of Montreal, University of Calgary) and PHAC recognized the lack of information regarding AMU, AMR, and the importance of improving AMS in the Canadian dairy sector. Together they decided to develop a surveillance system to fill the knowledge gap. This diverse group of researchers had expertise in epidemiology, antimicrobial resistance, dairy production medicine, surveillance system development, and public policy.

In order to initiate the development of the surveillance system a 5-year proposal was developed and funded by Dairy Farmers of Canada and Agriculture and Agri-food Canada, under the Dairy Research cluster 3 program, and by PHAC and the University of Prince Edward Island (UPEI). After the initial funding (2018–2022) the intention is to incorporate this system into CIPARS.

An Expert Panel (EP) was created to develop a farm-based surveillance framework for AMU, AMS, and AMR on dairy farms across Canada. The EP was composed of researchers from six Canadian universities (University of Prince Edward Island, University of Guelph, University of Saskatchewan, University of Montreal, University of Calgary, and Memorial University) and veterinary epidemiologists from the PHAC.

In the summer of 2018, members of the EP developed a draft of the surveillance framework. As part of the framework development, it was decided that the surveillance system should be deployed in five regions across Canada. These regions were the communities of Truro/Halifax in Nova Scotia, Montérégie region in Québec, London Middlesex in Ontario, Calgary-East in Alberta, and Fraser Valley in British Columbia, which are part of the sentinel sites from FoodNet Canada, a surveillance system focused on foodborne and waterborne diseases (25).

During the initial development phase of the surveillance framework, a Steering Committee (SC) was created, and the framework was sent to them for comments in January 2019. The SC was composed of relevant stakeholders from provincial and national milk boards (e.g., Dairy Farmers of Canada), veterinary organizations (e.g., Canadian Association of Bovine Veterinarians), PHAC, dairy herd improvement organizations and others. The role of the SC was to provide input on developing the surveillance framework for implementation in 2019 and ensure that the methods to collect farm samples and data were practical and sustainable. In addition, SC members were tasked with disseminating findings from the surveillance system to their respective organizations.

After the initial development of the framework, the EP and the SC, came together for a 2-day meeting whereby the framework was introduced and discussed. Suggestions were offered to improve the quality of data generated and introduce the surveillance system to the Canadian dairy industry. The information generated from this meeting was used to refine and finalize the surveillance framework. A final framework was ready for implementation in the spring of 2019.

For the implementation of the surveillance system, an operation committee was created. The operations committee was composed of all EP members, regional project managers, regional field workers, technicians and graduate students involved in the system. The role of the operations committee was to provide feedback on the operational issues through monthly meetings after the surveillance implementation and contribute to potential refinements of the surveillance system.

**Figure 1:**
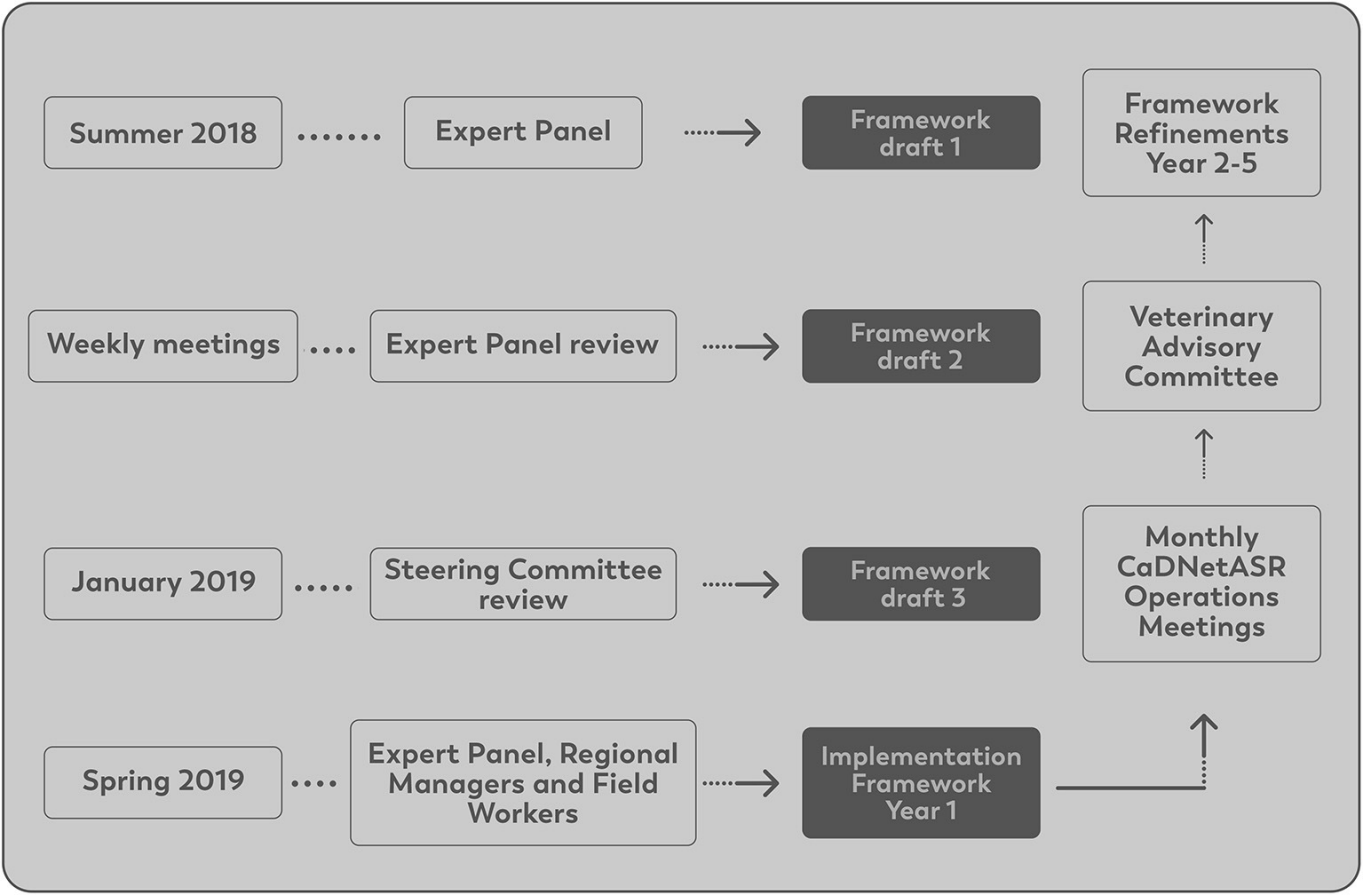
CaDNetASR framework development and implementation.

Each of the five regions had one regional project manager responsible for overseeing herd selection, the data collection and supervising the regional field workers. The regional field workers scheduled the farm visits and conducted the sampling based on the protocols provided. The surveillance system (CaDNetASR) was implemented in September 2019 and continued for 4 years in the first round of funding. The development and implementation of CaDNetASR is illustrated in Figure 1.

## CaDNetASR Surveillance Components

**Figure 2:**
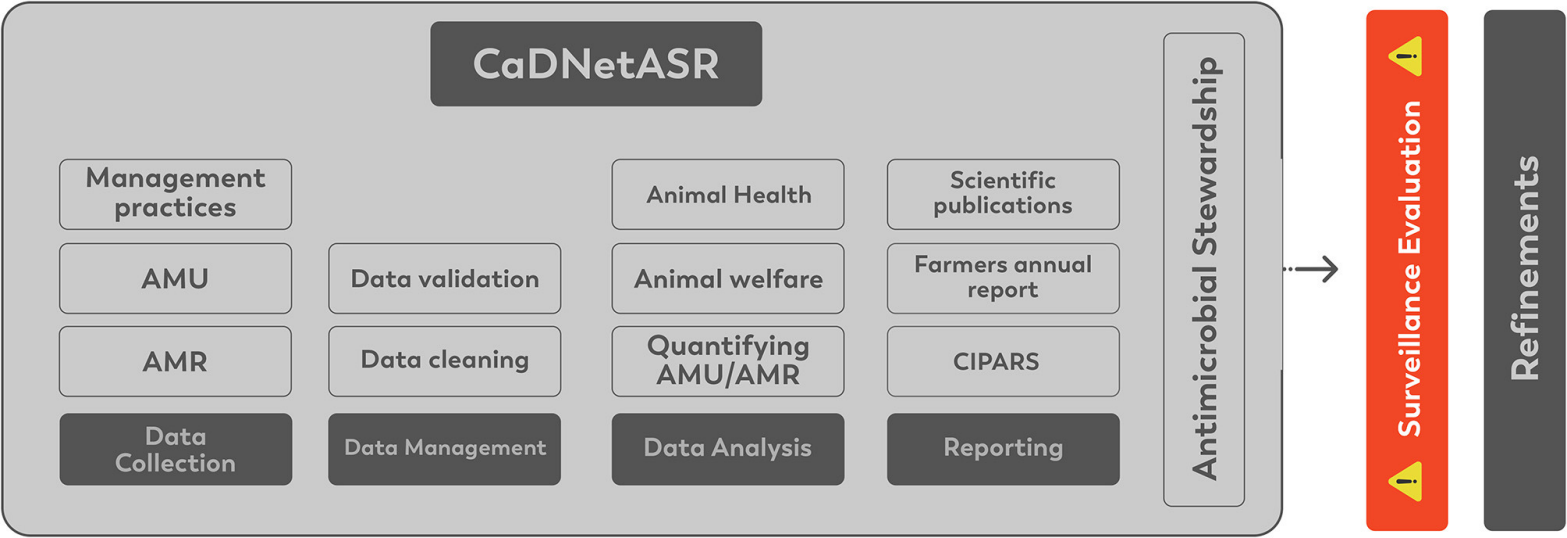
CaDNetASR surveillance system components.

The CaDNetASR surveillance includes all the critical components for AMR and AMU surveillance, collecting, analyzing, and reporting AMR and AMU in dairy herds at the farm level. The components of CaDNetASR are described below and are illustrated in Figure 2.

### Farm Enrollment

As the AMU stewardship was a key component in the surveillance system, the sample size was calculated to estimate an AMU rate with a precision of +/- 0.3 for various antimicrobials based on the assumption that 95% of the farms have AMU rates between 0.001 and 4 ADD/1,000 cows (26). Therefore, the goal was to select 30 farms from each of the five regions to participate in the research project. At implementation in 2019, a convenience sample of 144 dairy farms was enrolled. All regions enrolled 30 farms except Nova Scotia, where only 24 farmers agreed to participate. In 2020, three herds from British Columbia and one herd from Quebec dropped out of the program and were replaced with new herds. Farms should be representative of commercial dairy operations in each region. The following inclusion criteria were considered: (1) farms should be enrolled in ProAction/CQM (national mandatory certification program focused on several aspects of milk production) and DHI (dairy herd improvement organization responsible for milk recording, genetic evaluations and knowledge transfer in Canada); (2) minimum herd size of 50 animals except for Nova Scotia, that was minimum herd size of 40 animals; (3) raise their replacement heifers on-site; (4) Antimicrobial-free, organic or robotic herds should be enrolled proportional to their prevalence in a given region; (5) farmers should be willing to provide/share drug purchase information obtained from their veterinary clinics and feed mills. The only exclusion criteria were farms not planning to continue farming for the next 5 years. To protect the identity of participating farms, each farm was assigned an identifier, and only the regional project managers recorded which farm was linked to the study identifier to maintain anonymity. All producers signed an informed consent form explaining the project objectives and their role as participants, at the beginning of the 1st year, which was reviewed with them annually. The summary of demographic information for the dairy farms enrolled in CaDNetASR is presented in Table 1.

**Table 1:** Summary of demographic information from dairy farms enrolled in CaDNetASR during 2019 and 2020.

	**Province**				
**Characteristic**	**British Columbia**	**Alberta**	**Ontario**	**Quebec**	**Nova Scotia**
Farms enrolled	30	30	30	30	24
Herd size^*^ (mean)	175.3	170.4	159.8	86.3	101.1
% Free stall	100.0	96.6	87.1	21.4	62.5
% Tie-stall	0.0	3.3	9.7	74.2	37.5
% Other housing	0.0	0.1	3.2	4.4	0.0
Milking parlor	57.1	76.6	48.4	21.4	37.5
Robotic	42.9	23.4	41.9	12.9	16.7
Milking Pipeline	0.0	0.0	9.7	65.7	45.8
% Holstein	90.7	93.7	97.9	91.9	97.0
% Jersey	6.0	3.6	0.7	0.8	0.7
% Other breeds	3.3	2.7	1.4	7.3	2.3

* *Number of lactating cows*.

### Data Collection, Data Management, and Reporting

On-farm data collection included annual collection of fecal samples, a bulk tank milk sample (BTM), administration of questionnaires to collect herd management practices, AMU, and risk factor information for AMR related projects/questions. The main sections of the questionnaires are presented in Supplementary Tables 1,2 . Regional field workers collected pooled fecal samples from up to five pre-weaned calves, five breeding age heifers and five lactating cows and a single sample from the manure storage system by pooling from three to five different locations in that system. Standardized sampling kits designed by PHAC were sent to each regional project manager.

Samples were stored in a cooler with ice and sent to be processed at the central laboratory. Upon arrival at the laboratory, samples were processed for generic *E. coli, Campylobacter spp*., and *Salmonella spp*., in addition to preserving the raw sample following the protocol used by CIPARS (19). A 1 mL aliquot of each sample was saved for potential further processing. If there was growth on any of the three plates, then a single representative bacterial isolate was selected and stored. In 2019, a total of 560 fecal samples were collected and cultured. The proportion of samples positive for each target bacterial species were as follows: *E. coli*- 97.1% (544/560); *Campylobacter* spp.−63.8% (357/560); and *Salmonella* spp.−5.0% (28/560). In 2020, a total of 574 samples were collected and cultured. 94.4% (542/574), 49.1% (282/574), and 7.7% (44/574) of samples were positive for *E. coli, Campylobacter* spp. and *Salmonella* spp., respectively. The information is presented in Table 2. Susceptibility testing on the stored isolates was done using the broth microdilution system method (Sensititre, ThermoFisher, Mississauga). *E. coli* and *Salmonella* spp. were tested against 14 antimicrobials using the CMV2AGNF plate (27), and *Campylobacter* spp. was tested against eight antimicrobials using the CAMPY AST plate designed by the National Antimicrobial Resistance Monitoring System (28). All results were extracted to a Microsoft Excel (office 16) spreadsheet by the laboratory technicians and uploaded into the central digital platform.

**Table 2:** Proportion (%) of fecal samples positive for target bacteria processed in 2019^a^ and 2020^b^.

**Target bacteria**	**Calf**		**Heifer**		**Cow**		**Manure pit**	
	**2019**	**2020**	**2019**	**2020**	**2019**	**2020**	**2019**	**2020**
Generic *E. coli*	97.9	98.6	99.3	99.3	99.3	100.0	92.1	79.7
*Campylobacter* spp.	31.4	21.5	82.9	66.4	84.3	72.2	56.4	36.4
*Salmonella* spp.	3.6	3.5	2.1	4.9	2.9	4.9	11.4	17.5

*^a^A total of 140 samples were analyzed by each production phase and manure pit; ^b^A total of 144 samples were analyzed by each production phase and manure pit*.

During the initial phase of CaDNetASR, the garbage can audit (GCA) was implemented for a period of 6 months to quantify AMU. The farmers were advised to deposit all the empty antimicrobials vials (bottles, packages, and tubes) in the receptacles, which were placed strategically where antibiotics might be administered around the farm. The contents of the receptacles were collected and recorded by the regional field workers. In addition, the regional field workers collected information on the antimicrobial inventory at the beginning and the end of the GCA period. The quantities of each antimicrobial were later converted to dose-based metric developed for Canadian dairy cattle as published by Lardé et al. (29). For the following years, antimicrobial use will be estimated using veterinary clinic dispensing records. A Veterinary Advisory Committee (VAC) composed of three veterinarians was created to help understand how best to extract information from clinic electronic medical records. The surveillance components on AMU and AMR data are summarized in Table 3.

**Table 3:** Summary of the key activities of the CaDNetASR on-farm surveillance system.

**Data collection**	**Data management**	**Data analysis**	**Data reporting**	**Antimicrobial stewardship**
**AMR** Annual bulk tank milk and composite fecal samples from: • Pre-weaned calves • Breeding age heifers • Lactating cows • Manure storage	Samples are shipped to one central laboratory and cultured for: • Generic *E. coli* • *Campylobacter* spp. • *Salmonella* spp. • Antimicrobial susceptibility test (MIC) Freeze-dried isolates bank • The results from the laboratory are recorded and uploaded to the central digital platform. All the data is anonymized for privacy protection	Analysis of resistance profiles over time, regions, and sample types	• Annual report with summary AMR results and AMU benchmarking for farmers and veterinarians • Scientific publications • CaDNetASR results integrated with CIPARS reports (integrated surveillance data reporting AMU and AMR trends from animals and humans)	• Development of decision support charts and guidelines for efficient use of antimicrobials • Develop decision support tools and educational material highlighting the importance of the prudent use of antimicrobials • Target interventions on management practices where the use of antimicrobials can be done more responsibly (e.g., dry-cow treatment, udder infections, etc.)
**AMU** Annual collection of dispensing veterinary records	All AMU data are converted to the dose-based metric (DDD/DCD) and uploaded to the central digital platform after being validated by members of the operations committee. All the data is anonymized for privacy protection	Analysis of AMU converted to DDD and DCD/100 animals/year over time, regions, active ingredients, and administration routes		
**Questionnaire** Annual data collection on management practices (demographics, animal health, biosecurity, AMU)	Each regional field worker is responsible for recording the questionnaire information into a spreadsheet that is uploaded to the central digital platform after being validated by the regional managers.	The questionnaires will provide information on potential risk factors that can contribute to the development of AMR, which can impact animal health and animal welfare		

**Figure 3:**
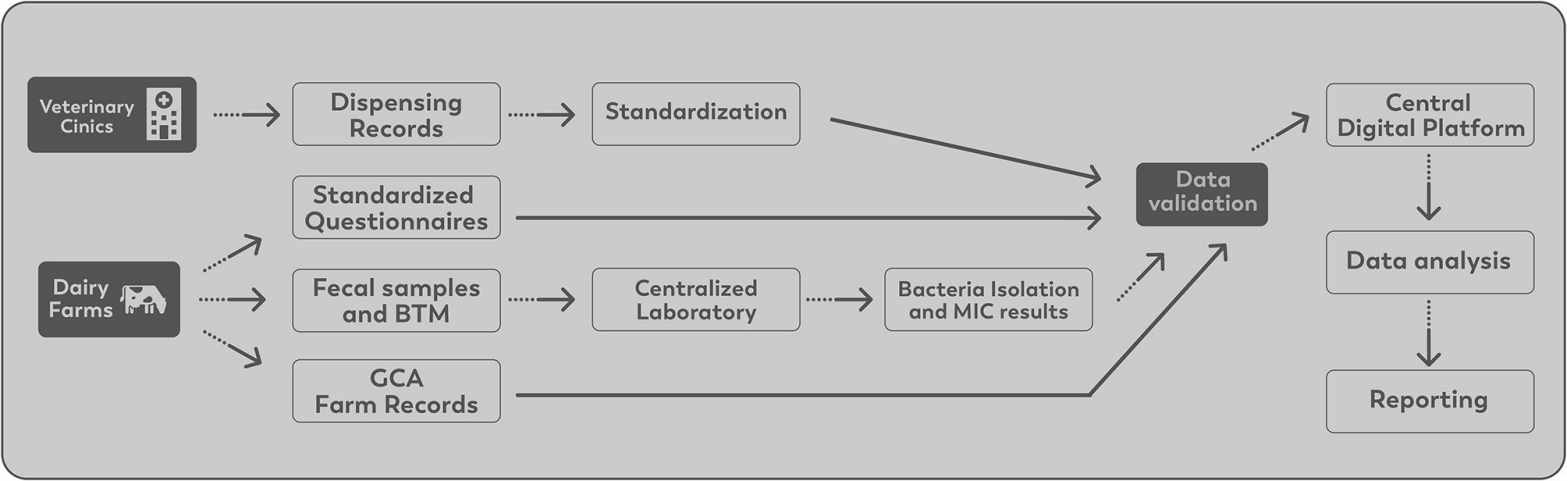
CaDNetASR communication policy and data flow.

Data are managed through a collaborative and integrated computer system developed to store the data generated by the surveillance system efficiently. All data are standardized, validated, and uploaded to the central digital platform. All information stored in the digital platform is protected by restricted access. The data flow is illustrated in Figure 3.

An important component for surveillance systems is knowledge dissemination. There is a diverse group of stakeholders interested in data regarding AMU and AMR in dairy cattle. These include veterinarians, academia, industry, policymakers, producers, government, public, among others. After each year, summary findings on AMU and AMR are being sent to participating producers and their veterinarians (Supplementary Figure 1). Reports include benchmarking data on AMU, which allow comparisons within participant farms. The report also includes a summary of AMR in the target pathogens. CIPARS publishes annual reports and will incorporate the dairy cattle data along with other animal species (e.g., pigs, poultry, and turkey). Peer-reviewed publications and abstracts for conferences are being prepared according to data availability.

## Discussion

There is increasing pressure on animal agriculture to justify the use of antimicrobials to treat and prevent infections in animals. Antimicrobial use is the main driver of resistance in target and non-target bacteria in food animals, which can potentially pass to humans via the food chain (30). In the United States, almost 70% of respondents from the general public believed that AMU in dairy cattle represented a moderate to high threat to human health (31). In another study in Canada, 28% of the respondents from the general public reported that they prefered not to consume products from animals raised with antimicrobials (32). The development of CaDNetASR provides AMR and AMU information for another major food animal production system in Canada.

Antimicrobial stewardship is a key factor for mitigating the effects of AMR (21) but changing how antimicrobials are used on farms can be challenging. To improve AMS in the food animal industries in Canada, all Medically Important Antimicrobials (MIAs) for veterinary use are sold by veterinary prescription only. Additionally, to support AMS by veterinarians, the CVMA launched the “SAVI” initiative (The Stewardship of Antimicrobials by Veterinarians Initiative). This initiative was supported by the government of Canada and the Canadian Agricultural Partnership. It consists of an electronic platform that has information on AMS and helps veterinary practitioners make informed decisions on AMU in their patients (33). CaDNetASR will support these initiatives by collecting and analyzing AMU and AMR and determining any changes that may be occurring.

AMS initiatives can have significant impacts on AMU and AMR on farms. For example, in the Netherlands there are compulsory and voluntary programs that affect AMS in farm animals, including dairy cattle. The RESET Mindset Model (34) was a stewardship strategy used in the Netherlands in the dairy sector aiming to limit the use of critically important antimicrobials and to ban the preventive use of antimicrobials as in blanket dry cow treatment. This model is a behavioral change intervention aimed at more rational use of antimicrobials by farmers and veterinarians and has proven to be effective at reducing AMU. These programs combined with new regulations have resulted in a 56% decrease in total AMU on participating farms between 2007 and 2012 (35, 36). In Switzerland, interventions targeting management practices on udder health, uterine health, and calf health were implemented on farms that were followed for 3 years. The implementation of these interventions provided knowledge for evidence-based decisions that contributes to better AMU stewardship (37).

Most dairy farms in Canada are in the provinces of Quebec and Ontario, and the production of fluid milk is regulated in Canada using a quota system. Federal and provincial organizations adjust quota to meet expected consumer demand. Milk produced in a province is frequently consumed within the province. Therefore, to ascertain AMR and AMU practices for Canadian dairy herds, it is necessary to conduct surveillance in as many provinces as possible. Each farm is visited annually for sample collection from three different age groups, which can aid in investigating AMR patterns in all stages of dairy production and may help target interventions where they are needed most. Additional data (herd demographic and farm management information) were collected on-farm using two questionnaires. All the information collected is standardized and stored in a central database. In the first 2 years, the questionnaires were administered using standardized spreadsheets that required manual data entry. In the process of validating these data, input errors were found, which had to be corrected. Automated processes for data entry are preferable to manual entry, and in future years, data will be uploaded from a hand-held device directly to a central database without the need for manual data entry.

The primary outcome of CaDNetASR is to inform the Canadian dairy industry, the general public, and policy decision makers on the level of AMU and AMR, and the impact that AMS practices have on AMU and AMR on Canadian dairy farms. Recently, 15 countries collecting AMU data at the farm level were identified (38). Among these countries, 12 have dairy surveillance programs monitoring AMU (Supplementary Table 3), and only seven of these countries collect and report AMU at the farm level. A major feature of CaDNetASR is that AMU data is collected at the farm level for dairy cattle. Farm level AMU data results in better estimates of AMU as it can account for the number of exposed animals, exposed time, and biomass on individual farms and allows for benchmarking, which can be used to compare high and low users of antimicrobials (38).

High quality estimates of AMU from surveillance programs are essential to provide reliable results. AMU estimates can be made from a variety of sources. In Denmark, for instance, there is a national, centralized database (VetStat) that collects AMU data at the herd level. The VetStat was implemented in 2000, and the program estimates AMU by collecting antimicrobial dispensing records from pharmacies, veterinarians, and feed mills for individual farms (39). In the Netherlands, estimation of farm level AMU started in 2004 with the implementation of MARAN (Monitoring of Antimicrobial Resistance and Antimicrobial Usage in Animals in the Netherlands). At the implementation, only a sample of farms was part of the program, and the experience gained with MARAN was used as a base for the development of a sectoral quality assuring system that collects AMU data nationally from the different animal sectors in Netherlands (38). In 2010, the Netherlands Veterinary Medicines Authority (SDa) was established to receive and centralize the AMU information from the sectoral systems (veterinary prescriptions) and from national sales (pharmaceutical industry). All the AMU information is reported annually through the MARAN program.

Since 2018, the Veterinary antimicrobial sales reporting system (VASR) system in Canada has provided an annual report regarding the sales of veterinary antimicrobials considered important for human medicine (40). The information gathered by the VASR system provides crude estimates of the amount of antimicrobials used in animals in the different agricultural production sectors. This information is adequate to estimate AMU on a national scale but is not precise enough to estimate AMU at the farm level (41).

Efforts in Canada to improve farm-level estimates of AMU are ongoing. One method that has been used is the GCA, which is considered the reference test for farm-level AMU estimates. GCAs are very labor intensive and time consuming, so other approaches for estimating AMU must be found. In Québec, a recent study investigated different methods of collecting AMU data at the farm level (42). GCA was used as reference method and were compared with information collected through veterinary invoices, information from the Amélioration de la Santé Animale au Québec (ASAQ) Program (Provincial Government), and farm treatment records. It is important to mention, that in Québec, almost 90% of the veterinary clinics providing antimicrobials to dairy farms, use the same office management software (Vet-Expert software), which facilitates data standardization (42). Veterinary invoices were found to have almost a perfect agreement with GCA and proved to be a reliable estimate of AMU. In the CaDNetASR system, the collection of veterinary clinics dispensing records was chosen to estimate farm-level AMU. This will demand standardization because of the variety of software packages used by veterinary clinics in Canada (other than the province of QC). To help with this process, 49 veterinary clinics that provided veterinary services, including sales of antimicrobials, to the 144 enrolled dairy herds were contacted and asked about their clinic software and how their AM sales were tracked. Responses from 23 clinics showed that only eight different electronic software systems were being used. Furthermore, there were also many differences in how sales were reported within each system. Consultations with the VAC helped CaDNetASR administrators understand the challenges associated with AMU data extraction from these different systems and to help determine the best approach to clinic engagement for data provision. Members of this group also provided preliminary herd-level dispensing data, which were helpful in the development of automated routines necessary for the standardization of dispensing record data. This approach to AMU data collection and estimation will improve the quality of the dispensing record data received by CaDNetASR.

AMU data collected by CaDNetASR, was transformed into a dose-based metric, to account for the different dosages among the different active ingredients. The dose-based metric divides the total amount of antimicrobial used (mg) by total animal weight and estimate daily dose for the antimicrobial (43). There is no perfect metric, and the choice of a metric to be used should be made based on the surveillance objectives. Ten countries monitoring AMU at farm level use dose-based metrics to quantify AMU (32) which allow for meaningful and comparable estimates of AMU within the different animal sectors (38). A specific dose-based metric was developed for dairy cattle in Canada (23), and it is being used to estimate AMU in the CaDNetASR (29).

In addition to the amount of AMU on farms it is important to determine which antimicrobial is used as well. Some antimicrobials are more important than others in treating infections in humans and their use in animal agriculture should be minimized and used only when other antimicrobials are known to be ineffective. The WHO publishes a regularly updated document, classifying the antimicrobials according to their human importance (44). In Canada, Health Canada's Veterinary Drugs Directorate (Government of Canada, 2009) has categorized the antimicrobials according to their importance in human and veterinary medicine (45). These classifications can provide meaningful information to be included in the AMS goals, aiming to decrease the usage of highly important antimicrobials for human medicine (46).

CaDNetASR is collecting AMR data from the following organisms: *Salmonella spp*., *Campylobacter spp., and E. coli*. These bacteria were selected because they are important zoonotic pathogens, where AMR is a concern or in the case of generic *E. coli*, it is thought to reflect the reservoir of resistance genes. These bacteria are monitored in other CIPARS' surveillance programs (27) and have been recommended by the European Food Safety Authority (EFSA) (47). By monitoring AMR in these target organisms, it may be possible to determine trends in resistance profiles. Ideally, after AMU interventions have been applied to surveillance farms, AMR in the target organisms will decrease.

Not all countries report AMR in the same organisms. Among the thirteen countries listed in Supplementary Table 4, only five provided information regarding AMR in bacterial isolates from dairy cattle in their national reports: Belgium, Denmark, Netherlands, Sweden, and United States. In the United States, NARMS monitors *Salmonella spp*., *Campylobacter spp., Enterococcus* spp., and the indicator *E. coli* from cecal samples of dairy cattle collected at the abattoir (48). In Belgium (FASFC), Denmark (DANMAP), and Sweden (SVARM), only MRSA *Staphylococcus aureus* is targeted for AMR surveillance in dairy cattle. The most common MRSA clone in production animals is the Livestock Associated MRSA (LA-MRSA), which has been associated with pig production (49). In Denmark and Sweden, the prevalence of LA-MRSA in dairy production remains low and it is not thought to be of concern in North America either (50, 51). In Canada, the MRSA in dairy production also has a limited occurrence. A study conducted in 91 herds across six provinces in Canada screened 1802 *Staphylococcus aureus* isolates for MRSA, and only one isolate was positive (0.05%) (52). For this reason, the inclusion of MRSA in CaDNetASR was not considered. In the Netherlands (MARAN), annual surveillance for ESBL-producing *E. coli* from cattle fecal samples is reported. After the 3rd year, CaDNetASR will be reporting recovery of ESBL-producing *E. coli* as well. Monitoring ESBL- producing enterobacteria is of critical importance as they pose a threat to human health (53). To the author's knowledge, CaDNetASR is the only surveillance system for dairy cattle that monitors AMR in enteric bacteria in different production phases and from manure storage.

Another important feature of the CaDNetASR system is the development of an isolate bank. All bacterial isolates will be freeze-dried and stored for future analysis. Although currently WGS is being done only for *Salmonella* spp. isolates, the idea is to expand to other isolates of interest, as it is anticipated that WGS will be routinely done in the future. The isolate bank will allow for the comparison of data from historical isolates to those collected in the future. In some European countries, WGS is being implemented gradually, and it will be mandatory after 2026 (47). The WGS data can be used as a complementary tool to the phenotypic AMR surveillance data and provide more information on the AMR epidemiology. Another new approach used for AMR detection is the use of metagenomics. Shotgun metagenomics allows for the detection of the entire bacterial community in a sample. If using traditional culture methods only cultivable organism will be detected and some important data may be missed (54). In the future, the inclusion of metagenomic approaches to characterize the resistome of a sample will improve the monitoring of the spread of resistance genes and the association between resistance from animals and humans.

## CaDNetASR Surveillance System Limitations

The development of a surveillance system requires an iterative process that will reduce data limitations and biases. Some of these limitations can be interpreted in the context of the main goals of the surveillance system. For instance, dairy farms were recruited by local veterinarians to participate in CaDNetASR. Therefore, the results from these farms should only be extrapolated to the study farms. Participating farmers might be more motivated and might have differing management practices and burdens of AMR compared to non-participating farms. According to the EFSA recommendations (47) samples should come from randomly selected epidemiological units to avoid sampling bias. CaDNetASR enrolled farms were not randomly selected, although, the samples collected within farms, were randomly selected from healthy animals, following the recommendations. Thus, it believed that findings can be extrapolated with caution to similar commercial operations. Data coverage is also a key factor that can affect the interpretation of surveillance results. Ideally monitoring would be conducted on as many farms as possible to obtain more precise results. Although CaDNetASR is not a full coverage system, it includes farms from five different provinces in Canada, and it could be used as a model to expand surveillance in the future.

The cross-sectional design implemented in CaDNetASR can bring disadvantages for supporting causal inferences, however, a major goal of the system is to benchmark AMR / AMU patterns across years and regions rather than making a causal inference. Three other major limitations can be considered for this surveillance system: (1) Yearly sampling. This sampling scheme will limit the possibility of tracking seasonal variability; however, each farm is sampled during the same season, allowing comparisons over time; (2) Sample type. CaDNetASR is based on pooled samples from three different ages of cattle and samples from two areas of the farm (calves, heifers, cows, manure pit and BTM. In the future, CaDNetASR will evolve to genomic methods, detecting pathogens and AMR genes. Pooled individual samples have been recommended, as it provided optimal results measuring AMR genes at herd level (55). But still, the surveillance system might miss resistant bacteria occurring in other environments in the farm (e.g., feed, water) (56, 57) which could lead to a low diagnostic sensitivity. However, the sampling scheme used in CaDNetASR includes three age groups, the manure pit and BTM, which will increase the chances of detecting antimicrobial resistant bacteria; and (3) Number of isolates. CaDNetASR has not established a required number of isolates to make inferences about the proportion of resistant bacteria. The initial years of CaDNetASR will provide the baseline trend information that will be used to develop sample size calculations for the ongoing surveillance.

Limitations can also occur in other two components of data collection in CaDNetASR: AMU and questionnaire information. AMU was initially estimated using a GCA system, which is time-consuming and prone to human errors. For this reason, all data were validated by each regional field worker to minimize errors. However, it is envisioned for the next years the AMU will be quantified using veterinary dispensing records. In Canada, all the antimicrobials are sold only with a veterinary prescription, thus, it is believed that veterinary dispensing records can provide a reliable estimation of AMU at farm level. Inaccurate results can arise from questionnaires when response bias occur in data collected. The questionnaires applied during the visits are long, which can demotivate the responders. However, to avoid that, the answers were entered by the regional field workers, that were also responsible to contact again the farmers to fill missing questions or to revise answers. Thus, this procedure is expected to reduce bias.

## Conclusions

In conclusion, the implementation and ongoing development of CaDNetASR are essential to guide AMS on dairy farms across Canada. It will also contribute to the Canadian program for AMR on animal health and public health. Finally, it will help stakeholders in the agricultural commodity groups to achieve more rational AMU on-farm, maintain and improve animal welfare, and support public health by diminishing AMR's burden.

## Data Availability Statement

The datasets presented in this article are not readily available because confidentiality and data ownership. Requests to access the datasets should be directed to mfonseca@upei.ca.

## Ethics Statement

The animal study was reviewed and approved by University of Prince Edward Island Research Ethics Board on March 7, 2019. Written informed consent was obtained from the owners for the participation of their animals in this study.

## Author Contributions

MF prepared the initial draft, figures, tables, and appendices. JS and LH contributed with conceptual development and writing of the manuscript. DL, JM, DR, SD, DK, DR, and HB critically revised the manuscript. All authors contributed to the article and approved the submitted version.

## Funding

This research was supported by a contribution from the Dairy Research Cluster 3 (Dairy Farmers of Canada and Agriculture and Agri-Food Canada) under the Canadian Agricultural Partnership AgriScience Program and the Public Health Agency of Canada.

## Conflict of Interest

The authors declare that the research was conducted in the absence of any commercial or financial relationships that could be construed as a potential conflict of interest.

## Publisher's Note

All claims expressed in this article are solely those of the authors and do not necessarily represent those of their affiliated organizations, or those of the publisher, the editors and the reviewers. Any product that may be evaluated in this article, or claim that may be made by its manufacturer, is not guaranteed or endorsed by the publisher.
