# Supplementary material for: Canadian Dairy Network for Antimicrobial Stewardship and Resistance (CaDNetASR): An On-Farm Surveillance System
Source: Front Vet Sci. 2022 Jan 12;8:799622. doi: 10.3389/fvets.2021.799622 (PMC8790291; doi:10.3389/fvets.2021.799622)

Supplementary material

Table S1. Main sections of the herd level questionnaires applied at enrollment in CaDNetASR surveillance

| Questionnaire 1 | Questionnaire 2 |
| --- | --- |
| - Breeds  - Herd inventory  - Non-dairy cattle species at the farm  - Milking system  - Type of housing for each production phase (pre-weaned calves, heifers, and lactating cows)  - Farm treatment records (if and how individual treatments are recorded at the farm)  - Veterinary services: number of veterinary visits for emergencies (e.g., sick animals), and number of preventive herd health visits  - Drug use protocols (written protocols for the most common infectious diseases)  - How the protocols are accessed (paper, electronically on a computer, electronically on a handheld device)  - Antimicrobial drugs sources  - Biosecurity (biosecurity practices, herd additions, and vaccination) | - Frequency of use of several antimicrobials’ products (injectables, intramammary for lactating cows, intramammary for dry cows, intra-uterine, topical, feed, water, and oral bolus)  - Reasons for AMU (reasons to use antimicrobials in different production phases to treat the most common infectious diseases)  - Frequency of cases of the most common infectious disease in each production phase |

Table S2. Main sections of the stewardship questionnaire applied at enrollment in CaDNetASR

| Stewardship questionnaire | |
| --- | --- |
| Dry-off procedure | Calf management |
| - Frequency of tests for SCC  - Use of teat sealant  - Proportion of cows receiving teat sealant at dry-off  - Use of SCC to decide the use of teat sealer  - SCC cut-off to make the decision to use teat sealant  - Proportion of cows receiving antimicrobials at dry-off  - Use of SCC to select animals that needs antimicrobials at dry off  - SCC cut off to make the decision to treat the animal  - Use of previous mastitis history to select animals to be treated at dry off  - Proportion of clinical mastitis treated with antimicrobials  - Proportion of clinical mastitis cases treated with intramammary antimicrobials  - Proportion of clinical mastitis cases treated with systemic antimicrobials  - Proportion of clinical mastitis cases treated with both intramammary and systemic antimicrobials | - Use of antimicrobials to treat calf diarrhea, and respiratory disease  - Clinical signs considered to make the decision to treat the animal  - Use of wasted milk to feed the calves |

Table S3. Modified farm-level AMU surveillance systems for dairy cattle identified by Sanders et al.

| Country | AMU Surveillance for dairy cattle | Collects AMU at farm-level | Report AMU at farm-level | Coverage | AMU metrics |
| --- | --- | --- | --- | --- | --- |
| Austria | PHAROS | Yes | No | Full sector | Dose-based |
| Belgium | AB Register/BIGAME | Yes | Yes | Partial sector | Weight-based/Dose-based |
| Canada* | CIPARS/CaDNetASR | Yes | Yes | Sample | Dose-based |
| Czech Republic | DLN cattle | Yes | Yes | Sample | Weight-based |
| Denmark | VetStat | Yes | Yes | Full sector | Dose-based |
| Germany | VetCAb-ID/VetCAb(-S) | Yes | Yes | Sample | Count-based |
| Italy | ClassyFarm | Yes | Yes | Sample | Dose-based |
| Netherlands | SQS\|SDa | Yes | Yes | Full sector | Dose-based |
| Norway | VetReg | Yes | No | Full sector | Weight-based |
| Spain | NDVAP | Yes | No | Full sector | Weight-based |
| Sweden | SBA | Yes | No | Full sector | Weight-based |
| Switzerland | IS ABV | Yes | Yes | Full sector | Dose-based/Count-based |
| United States** | No | No | No | N/A | N/A |

*Not included in Sanders et al.

**No AMU at farm-level, but it was included to allow comparison in North America

Table S4. AMR surveillance systems including data collection and report on dairy cattle

| Country^a^ | AMR surveillance food-producing animals | AMR Surveillance for dairy cattle | Type of Surveillance | Dairy Cattle subcategory | Material | Frequency of sampling | Target bacteria | MIC interpretation |
| --- | --- | --- | --- | --- | --- | --- | --- | --- |
| AU | AURES | No | N/A | N/A | N/A | N/A | N/A | N/A |
| BE | FASFC | Yes | Active | Cows | Pool of nasal swabs | Every three years | MRSA *Staphylococcus aureus* | EUCAST |
| CA | CIPARS | Yes (CaDNetASR) | Active | Calves/Heifers/Cows/Manure storage | Feces/Bulk tank milk | Annual | *E. coli/Salmonella* spp.*/Campylobacter* spp. | CLSI |
| CZ | No | No | N/A | N/A | N/A | N/A | N/A | N/A |
| DK | DANMAP | Yes | Active | Cows | Composite milk of 5 cows | First report on 2019 | MRSA *Staphylococcus aureus* | EUCAST |
| GE | GERMVET | No | N/A | N/A | N/A | N/A | N/A | N/A |
| IT | ITAVARM -Reported only in 2003 | No | N/A | N/A | N/A | N/A | N/A | N/A |
| NL | NethMap-MARAN | Yes | Active | Cows | Feces | Annual | ESBL-producing *E. coli* | EUCAST |
| NO | NORM-VET | No | N/A | N/A | N/A | N/A | N/A | N/A |
| SP | VAV - last report 2005 | No | N/A | N/A | N/A | N/A | N/A | N/A |
| SW | SVARM | Yes | Active | Cows | Milk | Annual | MRSA *Staphylococcus aureus* | EUCAST |
| SZ | No information retrieved | No information retrieved | N/A | N/A | N/A | N/A | N/A | N/A |
| US | NARMS | Yes | Active | Cows | Cecal sample | Annual | *E. coli/Salmonella* spp.*/Campylobacter* spp*./Enterococcus* spp. | CLSI |

^a^ AU: Austria, BE: Belgium, CA: Canada, CZ: Czech Republic, DK: Denmark, GE: Germany, IT: Italy, NL: Netherlands, NO: Norway, SP: Spain, SW: Sweden, SZ: Switzerland, US: United States; ^b^ N/A: Not applicable

Figure S1. CaDNetASR annual reporting summarizing the findings at each farm. The first section of the report estimates the AMU in DDD/100 animals/year and benchmark the farm among the other farms enrolled in the program. It is also provided a table classifying the most common antimicrobials used at dairy farms according to their importance in human health. The last part of the report presents the results on generic *E. coli* susceptibility in each of the production phases and manure pit.


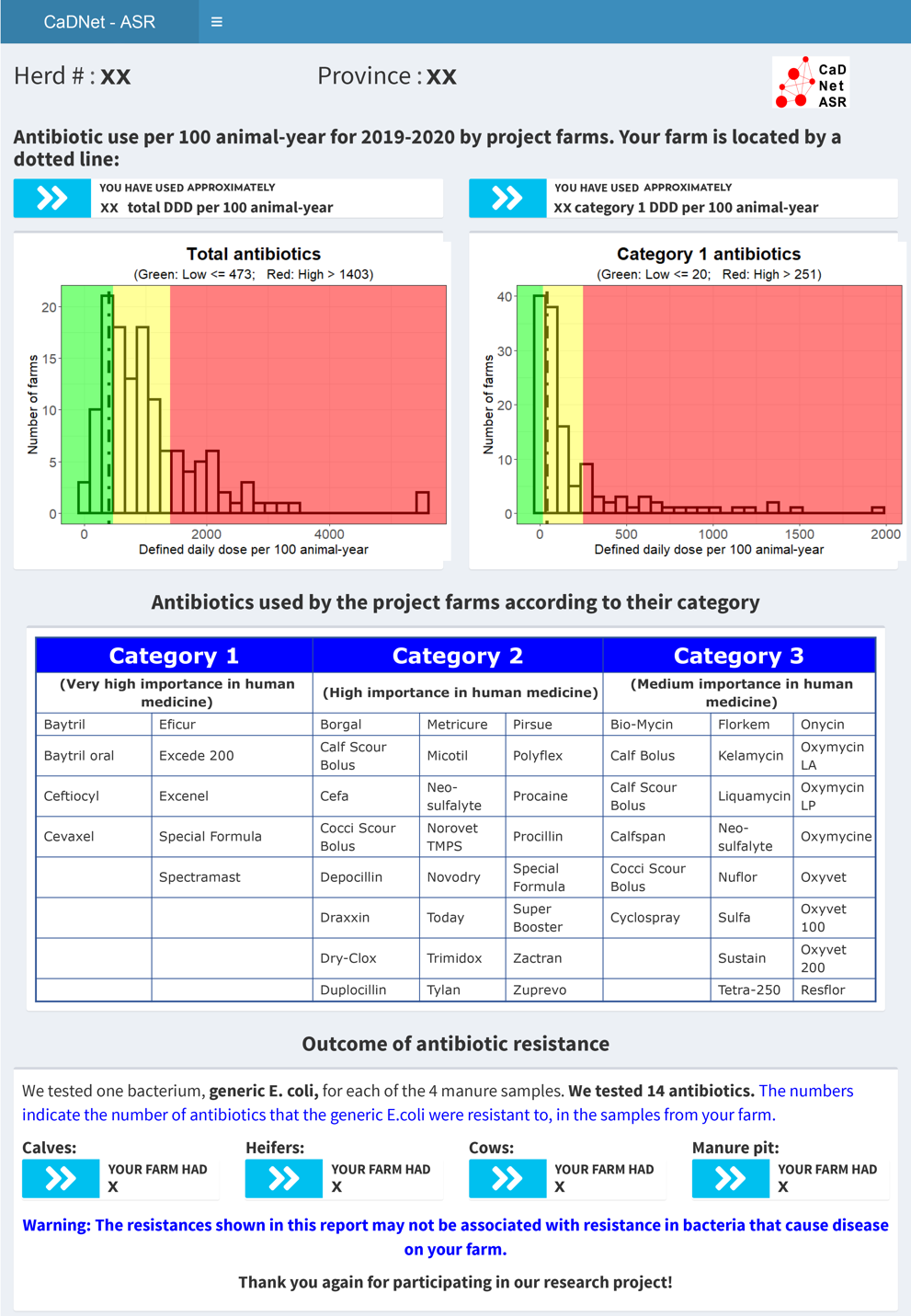

Supplement: Supplementary file 1 [file Table_1.DOCX]
